# Supplementary material for: Comparative analysis of four Zantedeschia chloroplast genomes: expansion and contraction of the IR region, phylogenetic analyses and SSR genetic diversity assessment
Source: PeerJ. 2020 May 22;8:e9132. doi: 10.7717/peerj.9132 (PMC7247528; doi:10.7717/peerj.9132)
Supplement: Table S1 [file peerj-08-9132-s001.pdf]

Table S1 The genes which contein introns in four *Zantedeschia* species.

**Gene**	**Location**	**Exon1(bp)**				**Intron1(bp)**				**Exon2(bp)**				**Intron2(bp)**				**Exon3(bp)**			
		Z. reh	***Z.odo***	***Z. ell***	***Z.aet***	***Z. reh***	***Z.odo***	***Z. ell***	***Z.aet***	***Z. reh***	***Z.odo***	***Z. ell***	***Z.aet***	***Z. reh***	***Z.odo***	***Z. ell***	***Z.aet***	***Z. reh***	***Z.odo***	***Z. ell***	***Z.aet***
atpF	LSC	401	402	401	401	844	897	844	862	145	145	145	145	/	/	/	/	/	/	/	/
clpP	LSC		1277	/	1274	/	836	/	849		71	/	36	/	795	/	653	/	1348	/	36
ndhA	LSC	553	553	553	539	1077	655	1077	1084	539	539	539	553	/	/	/	/	/	/	/	/
ndhB	LSC	777	756	756	777	661	1080	661	678	756	779	777	756	/	/	/	/	/	/	/	/
petB	LSC	6	6	6	6	56	678	54	56	642	642	642	642	/	/	/	/	/	/	/	/
petD	LSC	8	8	8	8	733	802	733	719	475	475	475	470	/	/	/	/	/	/	/	/
rpl16	LSC	399	399	399	399	1020	724	1008	1116	9	9	9	9	/	/	/	/	/	/	/	/
rpl2	LSC	428	370	428	382	666	661	666	661	376	429	376	434	/	/	/	/	/	/	/	/
rpoC1	LSC	1629	1620	1629	1620	737	743	735	742	459	432	459	453	/	/	/	/	/	/	/	/
rps16	IR	197	197	197	197	1161	1370	1180	1226	40	40	40	40	/	/	/	/	/	/	/	/
rps18	IR	24	/	24	23	147	/	219	30	282	/	282	295	/	/	/	/	/	/	/	/
ycf3	IR	1178	1138	1178	1148	708	746	710	748	124	124	124	124	795	755	795	765	124	124	124	1102
ycf68	IR	303	/	42	294	33	/	31	31	42	/	303	294	/	/	/	/	/	/	/	/
