# Supplementary material for: Comparative analysis of four Zantedeschia chloroplast genomes: expansion and contraction of the IR region, phylogenetic analyses and SSR genetic diversity assessment
Source: PeerJ. 2020 May 22;8:e9132. doi: 10.7717/peerj.9132 (PMC7247528; doi:10.7717/peerj.9132)
Supplement: Table S2 [file peerj-08-9132-s002.pdf]

Table S2. SSR candidates of the *Zantedeschia* chloroplast genome.

ID	*Z. odorata*			*Z. elliottiana*			*Z. aethiopica*			*Z. rehmannii*		
	**Repeat Motif**	**start**	**Region**	**Repeat Motif**	**start**	**Region**	**Repeat Motif**	**start**	**Region**	**Repeat Motif**	**start**	**Region**
1	(T)12	113	LSC	(TA)21	1460	LSC	(T)11	113	LSC	(TA)14	1287	LSC
2	(TA)5	2219	LSC	(C)10	5192	LSC	(TA)6	1603	LSC	(A)10	4020	LSC
3	(A)10	4957	LSC	(AT)17	5461	LSC	(C)12	5351	LSC	(T)11	5161	LSC
4	(C)12	6001	LSC	(TAAA)3	5550	LSC	(TAAA)3	5626	LSC	(TA)6	5307	LSC
5	(A)11	7686	LSC	(AT)5	7318	LSC	(A)10	8160	LSC	(A)10	6535	LSC
6	(A)10	9252	LSC	(A)13	9123	LSC	(A)12	8963	LSC	(AT)5	7067	LSC
7	(A)12	10047	LSC	(T)11	9427	LSC	(TA)9	9407	LSC	(A)11	8073	LSC
8	(A)14	11328	LSC	(A)10	9978	LSC	(A)13	10128	LSC	(A)12	8874	LSC
9	(A)10	12561	LSC	(A)10	11178	LSC	(T)12	11439	LSC	(A)11	12751	LSC
10	(T)12	12678	LSC	(T)10	11295	LSC	(A)10	15327	LSC	(A)11	13977	LSC
11	(A)10	16133	LSC	(A)10	12952	LSC	(A)10	15708	LSC	(A)14	14912	LSC
12	(A)17	16600	LSC	(A)11	14177	LSC	(T)10	16613	LSC	(A)11	15692	LSC
13	(A)11	17384	LSC	(A)10	15116	LSC	(TTA)4	16650	LSC	(T)10	16598	LSC
14	(T)10	18290	LSC	(T)10	16790	LSC	(T)11	17468	LSC	(TTA)4	16690	LSC
15	(T)11	19124	LSC	(TTA)4	16800	LSC	(T)14	19723	LSC	(T)10	17494	LSC
16	(T)13	21376	LSC	(T)10	17638	LSC	(TA)5	21102	LSC	(AT)5	18443	LSC
17	(TA)5	22754	LSC	(AT)5	18587	LSC	(TC)5	24108	LSC	(TA)5	21200	LSC
18	(TC)5	25768	LSC	(TA)5	21344	LSC	(ATAAT)4	28606	LSC	(T)10	23500	LSC
19	(A)10	30501	LSC	(T)10	23644	LSC	(T)12	29171	LSC	(TC)5	24213	LSC
20	(T)10	30775	LSC	(TC)5	24357	LSC	(TTTA)3	30421	LSC	(AATAT)3	28685	LSC
21	(T)10	31431	LSC	(T)14	28771	LSC	(A)18	30561	LSC	(A)13	28967	LSC
22	(A)13	32172	LSC	(AATAT)4	28890	LSC	(T)10	30822	LSC	(T)14	29213	LSC
23	(A)11	33305	LSC	(A)12	29119	LSC	(A)10	31706	LSC	(TCAA)4	30085	LSC
24	(TA)7	33923	LSC	(T)11	29379	LSC	(TA)5	32323	LSC	(A)10	30754	LSC
25	(AAT)5	33988	LSC	(A)10	30320	LSC	(TAA)5	23350	LSC	(TTTA)3	31203	LSC
26	(AT)6	34019	LSC	(T)10	30437	LSC	(A)10	33547	LSC	(A)14	31342	LSC
27	(AT)5	34738	LSC	(A)10	30907	LSC	(T)11	34006	LSC	(AT)19	33091	LSC

28	(TA)5	38107	LSC	(TTTA)3	31356	LSC	(TA)8	36751	LSC	(AT)8	33990	LSC
29	(T)10	38927	LSC	(A)15	31495	LSC	(T)10	37575	LSC	(T)10	34395	LSC
30	(A)10	40167	LSC	(C)10	32682	LSC	(A)12	37953	LSC	(T)12	34744	LSC
31	(A)11	47173	LSC	(AT)12	33263	LSC	(A)10	39042	LSC	(T)11	38289	LSC
32	(A)10	47427	LSC	(AT)8	33380	LSC	(T)10	45034	LSC	(GAAGA)3	38420	LSC
33	(A)10	47645	LSC	(T)11	34891	LSC	(A)13	46081	LSC	(A)11	39731	LSC
34	(TA)7	49314	LSC	(T)10	38438	LSC	(A)10	46337	LSC	(A)17	46762	LSC
35	(TA)5	49320	LSC	(GAAGA)4	38559	LSC	(A)10	46555	LSC	(A)10	47022	LSC
36	(ATA)5	49350	LSC	(A)10	38884	LSC	(ATA)4	46580	LSC	(ATA)4	47276	LSC
37	(ATA)6	49381	LSC	(A)10	39641	LSC	(A)10	47250	LSC	(A)14	47563	LSC
38	(A)12	51101	LSC	(A)19	46652	LSC	(T)10	48072	LSC	(A)12	47938	LSC
39	(T)10	53941	LSC	(A)10	46914	LSC	(TA)6	48273	LSC	(T)11	48789	LSC
40	(T)12	55002	LSC	(ATA)4	47164	LSC	(T)11	48815	LSC	(TA)16	48961	LSC
41	(A)11	57200	LSC	(A)11	47448	LSC	(A)16	50110	LSC	(ATTT)3	49518	LSC
42	(TA)6	61128	LSC	(T)10	48646	LSC	(T)10	50909	LSC	(A)13	50531	LSC
43	(TA)10	61158	LSC	(TA)11	48787	LSC	(T)10	52835	LSC	(A)15	51036	LSC
44	(A)12	62742	LSC	(ATTT)3	49438	LSC	(T)10	53910	LSC	(TTTTA)3	51426	LSC
45	(TC)5	67179	LSC	(TA)5	49925	LSC	(AT)5	59462	LSC	(T)10	51835	LSC
46	(T)12	70663	LSC	(A)14	50446	LSC	(TA)5	59798	LSC	(ATTAT)3	51880	LSC
47	(T)10	70990	LSC	(A)14	50952	LSC	(TA)5	59880	LSC	(TA)5	51980	LSC
48	(A)10	75206	LSC	(TTTTA)3	51341	LSC	(TA)6	59900	LSC	(TA)5	52889	LSC
49	(A)12	76097	LSC	(T)11	51750	LSC	(GAT)4	61167	LSC	(T)10	54327	LSC
50	(A)11	76589	LSC	(TA)5	51800	LSC	(A)11	61783	LSC	(T)10	54835	LSC
51	(T)10	76955	LSC	(TA)5	51890	LSC	(AAAT)3	63657	LSC	(T)14	58758	LSC
52	(TA)17	77836	LSC	(TA)5	51990	LSC	(AATA)3	63688	LSC	(TTCC)3	64620	LSC
53	(TA)6	77854	LSC	(TA)6	52800	LSC	(TC)5	66126	LSC	(A)11	67664	LSC
54	(A)11	81009	LSC	(T)10	53798	LSC	(AATG)3	66736	LSC	(TC)5	67685	LSC
55	(G)13	81276	LSC	(T)10	54798	LSC	(T)10	68335	LSC	(AATG)3	68337	LSC
56	(T)10	86123	LSC	(TTCC)3	64110	LSC	(TTGA)3	70845	LSC	(TCTT)3	68599	LSC
57	(T)10	90000	LSC	(TATAA)3	65071	LSC	(CTAGAG)3	72887	LSC	(T)11	69878	LSC
58	(T)15	90246	LSC	(A)11	67141	LSC	(AAT)4	73751	LSC	(AT)5	70193	LSC

59	(A)10	90813	IR	(TC)5	68120	LSC	(A)10	74590	LSC	(TTGA)3	72505	LSC
60	(T)10	102955	IR	(AATG)3	67814	LSC	(T)11	75322	LSC	(TA)6(TA)6	74156	LSC
61	(T)11	104430	IR	(TCTT)3	67890	LSC	(T)10	75571	LSC	(ATAAAT)3	74411	LSC
62	(T)10	104569	IR	(T)13	69355	LSC	(A)10	75968	LSC	(A)11	77360	LSC
63	(GAA)13	116600	IR	(TTGA)3	71997	LSC	(T)10	76337	LSC	(TA)5	77430	LSC
64	(GAG)6	118046	IR	(T)10	72641	LSC	(A)10	76789	LSC	(TATAG)3	77500	LSC
65	(A)11	118573	IR	(TA)6	73651	LSC	(TA)5	77207	LSC	(TA)5	77577	LSC
66	(A)10	119604	IR	(TA)6	73990	LSC	(A)10	80483	LSC	(AT)7	77600	LSC
67	(A)12	119848	IR	(ATAAAT)3	73978	LSC	(G)12	80687	LSC	(TA)9	77680	LSC
68	(A)10	120244	IR	(TTCCA)3	75243	LSC	(CATTCC)3	83347	LSC	(TATAG)4	77790	LSC
69	(T)11	122413	IR	(TATAG)3	76994	LSC	(T)10	85487	LSC	(TA)5	77836	LSC
70	(T)10	129971	SSC	(TA)5	77134	LSC	(T)10	86197	LSC	(AT)6	78900	LSC
71	(T)10	130295	SSC	(TTATA)3	78880	LSC	(T)10	87101	LSC	(TATAT)3	80790	LSC
72	(C)11	133477	SSC	(G)10	80611	LSC	(TCTA)3	87498	LSC	(A)10	81004	LSC
73	(T)10	137146	SSC	(A)11	81403	LSC	(T)15	89625	LSC	(G)12	81278	LSC
74				(TCTATA)3	84342	LSC	(A)10	90197	IR	(A)10	82072	LSC
75				(T)20	85541	LSC	(TATG)3	91599	IR	(TCTATA)3	85010	LSC
76				(AAAT)3	86089	LSC	(GTATTA)3	94394	IR	(T)15	86209	LSC
77				(TCTA)3	87308	LSC	(CAT)4	96740	IR	(AAAT)3	86753	LSC
78				(AT)6	88781	IR	(GGTTTA)3	96997	IR	(TCTA)3	87984	LSC
79				(T)15	89242	IR	(AT)	100183	IR	(T)12	89918	LSC
80				(A)10	90099	IR	(A)12	100574	IR	(A)10	90151	IR
81				(AT)5	92059	IR	(T)10	102292	IR	(A)10	90764	IR
82				(AT)5	92159	IR	(T)11	103770	IR	(CGC)4	93739	IR
83				(T)10	96196	IR	(T)10	103909	IR	(T)10	96669	IR
84				(TA)6	98619	IR	(AATAT)3	115212	IR	(AT)6	100286	IR
85				(AT)6	99885	IR	(GAA)13	116247	IR	(AT)9	100457	IR
86				(AT)11	100056	IR	(A)11	118235	IR	(T)10	102307	IR
87				(T)10	101925	IR	(A)10	119266	IR	(T)10	103213	IR
88				(T)10	102831	IR	(A)12	119510	IR	(T)10	103915	IR
89				(T)10	103533	IR	(AAAAAG)3	119950	IR	(AATACA)3	115531	IR

90	(A)12	116485	IR	(ATAA)3	120959	IR	(A)12	116868	IR
91	(TGA)5	117059	IR	(TAAAT)3	121000	IR	(TGA)4	117433	IR
92	(A)10	117891	IR	(AT)5	121134	IR	(A)10	118265	IR
93	(A)11	118016	IR	(TA)5	121288	IR	(A)11	118405	IR
94	(A)11	118238	IR	(T)11	121959	IR	(A)11	118627	IR
95	(A)13	119628	IR	(T)10	122179	SSC	(A)13	120026	IR
96	(AT)5	120296	IR	(ATCA)3	123441	SSC	(AT)5	120637	IR
97	(ATAA)3	120759	IR	(AT)5	123653	SSC	(AT)5	120770	IR
98	(TAAAT)3	121789	IR	(AT)8	123804	SSC	(ATAA)3	121163	IR
99	(AT)6	120924	IR	(T)10	123977	SSC	(TAAAT)3	121250	IR
100	(TA)11	121061	IR	(ATAA)3	124212	SSC	(ATAA)5	121350	IR
101	(A)10	121412	IR	(AAT)4	124230	SSC	(AT)5	121445	IR
102	(T)12	121741	IR	(TA)6	124439	SSC	(TA)10	121475	IR
103	(AT)6	125876	IR	(G)11	125732	SSC	(T)12	122146	IR
104	(TA)5	126111	IR	(T)10	126843	SSC	(AT)6	126281	IR
105	(AT)5	126220	IR	(CTTT)3	126888	SSC	(AACAT)3	126427	IR
106	(AT)5	126280	IR	(A)12	128431	SSC	(A)10	126759	IR
107	(TA)5	126399	IR	(A)10	128932	SSC	(ATCA)3	128155	IR
108				(A)10	129252	SSC	(AT)5	128364	IR
109				(AT)6	132588	SSC	(TCTTA)3	128990	SSC
110				(TATTTA)3	133347	SSC	(AAAT)3	131834	SSC
111							(T)12	132443	SSC
112							(AAAG)3	133966	SSC
113							(C)11	135138	SSC
114							(AATA)3	136054	SSC
115							(TATT)4	136377	SSC
116							(AT)6	136713	SSC
117							(TTGA)3	136921	SSC
118							(T)10	138320	SSC
119							(AT)5	138526	SSC
120							(AT)6	138796	SSC
