# Supplementary material for: Comparative analysis of four Zantedeschia chloroplast genomes: expansion and contraction of the IR region, phylogenetic analyses and SSR genetic diversity assessment
Source: PeerJ. 2020 May 22;8:e9132. doi: 10.7717/peerj.9132 (PMC7247528; doi:10.7717/peerj.9132)
Supplement: Table S3 — 0 indicates that the gene that does not exist in the species. *2 indicates that two copies exist in the species. [file peerj-08-9132-s003.pdf]

Table S3 gene list of the four species with annotation.

genes				annotation
Z.aethiopica	*Z.odorata*	*Z. rehmannii*	*Z.elliottiana*	
atpA	atpA	atpA	atpA	ATP synthase CF1 alpha subunit
atpB	atpB	atpB	atpB	ATP synthase CF1 beta subunit
atpE	atpE	atpE	atpE	ATP synthase CF1 epsilon subunit
atpF	atpF	atpF	atpF	ATP synthase CF0 B subunit
atpH	atpH	atpH	atpH	ATP synthase CF0 C subunit
atpI	atpI	atpI	atpI	ATP synthase CF0 A subunit
ccsA	ccsA	ccsA	ccsA	cytochrome c biogenesis protein
cemA	cemA	cemA	cemA	envelope membrane protein
clpP	clpP	clpP	clpP	ATP-dependent protease proteolytic subunit
matK	matK	matK	matK	maturase K
ndhA	ndhA*2	ndhA*2	ndhA*2	NADH dehydrogenase subunit 1
ndhB*2	ndhB*2	ndhB*2	ndhB*2	NADH dehydrogenase subunit 2
ndhC	ndhC	ndhC	ndhC	NADH dehydrogenase subunit 3
ndhD	ndhD	ndhD	ndhD	NADH dehydrogenase subunit 4
ndhE	ndhE	ndhE*2	ndhE*2	NADH dehydrogenase subunit 4L
ndhF	ndhF	ndhF	ndhF	NADH dehydrogenase subunit 5
ndhG	ndhG	ndhG*2	ndhG*2	NADH dehydrogenase subunit 6
ndhH	ndhH*2	ndhH*2	ndhH*2	NADH dehydrogenase subunit 7
ndhI	ndhI*2	ndhI*2	ndhI*2	NADH dehydrogenase 18kD subunit
ndhJ	ndhJ	ndhJ	ndhJ	NADH dehydrogenase subunit J
ndhK	ndhK	ndhK	ndhK	NADH dehydrogenase subunit K
petA	petA	petA	petA	component of cytochrome b6/f complex
petB	petB	petB	petB	cytochrome b6
petD	petD	petD	petD	cytochrome b6/f complex subunit IV
petG	petG	petG	petG	cytochrome b6/f complex subunit V
petL	petL	petL	petL	cytochrome b6/f complex subunit VI
petN	petN	petN	petN	cytochrome b6/f complex subunit VIII
psaA	psaA	psaA	psaA	photosystem I P700 chlorophyll a apoprotein A1
psaB	psaB	psaB	psaB	photosystem I P700 chlorophyll a apoprotein A2
psaC	psaC	psaC	psaC	photosystem I subunit VII
psaI	psaI	psaI	psaI	PSI reaction center subunit VIII
psaJ	psaJ	psaJ	psaJ	PSI reaction center subunit IX
psbA	psbA	psbA	psbA	photosystem II protein D1
psbB	psbB	psbB	psbB	photosystem II 47 kDa protein
psbC	psbC	psbC	psbC	photosystem II 44 kDa protein
psbD	psbD	psbD	psbD	photosystem II protein D2
psbE	psbE	psbE	psbE	photosystem II protein V
psbF	psbF	psbF	psbF	photosystem II protein VI
psbH	psbH	psbH	psbH	photosystem II phosphoprotein H
psbI	psbI	psbI	psbI	photosystem II protein I
psbJ	psbJ	psbJ	psbJ	photosystem II protein J
psbK	psbK	psbK	psbK	photosystem II protein K
psbL	psbL	psbL	psbL	photosystem II protein L
psbM	psbM	psbM	psbM	photosystem II protein M
psbN	psbN	psbN	psbN	photosystem II protein N
psbT	psbT	psbT	psbT	photosystem II protein T
psbZ	psbZ	psbZ	psbZ	photosystem II protein Z
rbcL	rbcL	rbcL	rbcL	ribulose-1,5-bisphosphate carboxylase/oxygenase large subunit
rpl14	rpl14	rpl14	rpl14	ribosomal protein L2
rpl16	rpl16	rpl16	rpl16	ribosomal protein L14

rpl2*2	rpl2*2	rpl2*2	rpl2*2	ribosomal protein L16
rpl20	rpl20	rpl20	rpl20	ribosomal protein L20
rpl22	rpl22	rpl22	rpl22	ribosomal protein L22
rpl23*2	rpl23*2	rpl23*2	rpl23*2	ribosomal protein L23
rpl32	rpl32	rpl32	rpl32	ribosomal protein L32
rpl33	rpl33	rpl33	rpl33	ribosomal protein L33
rpl36	rpl36	rpl36	rpl36	ribosomal protein L36
rpoA	rpoA	rpoA	rpoA	RNA polymerase alpha subunit
rpoB	rpoB	rpoB	rpoB	RNA polymerase beta subunit
rpoC1	rpoC1	rpoC1	rpoC1	RNA polymerase beta' subunit
rpoC2	rpoC2	rpoC2	rpoC2	RNA polymerase beta' subunit
rps11	rps11	rps11	rps11	ribosomal protein S2
rps12*2	rps12*2	rps12*2	rps12*2	ribosomal protein S3
rps14	rps14	rps14	rps14	ribosomal protein S4
rps15*2	rps15*2	rps15*2	rps15*2	ribosomal protein S7
rps16	rps16	rps16	rps16	ribosomal protein S8
rps18	rps18	rps18	rps18	ribosomal protein S11
rps19	rps19	rps19	rps19*2	ribosomal protein S12
rps2	rps2	rps2	rps2	ribosomal protein S14
rps3	rps3	rps3	rps3	ribosomal protein S15
rps4	rps4	rps4	rps4	ribosomal protein S16
rps7*2	rps7*2	rps7*2	rps7*2	ribosomal protein S18
rps8	rps8	rps8	rps8	ribosomal protein S19
rrn16*2	rrn16*2	rrn16*2	rrn16*2	16S ribosomal RNA
rrn23*2	rrn23*2	rrn23*2	rrn23*2	23S ribosomal RNA
rrn4.5*2	rrn4.5*2	rrn4.5*2	rrn4.5*2	4.5S ribosomal RNA
rrn5*2	rrn5*2	rrn5*2	rrn5*2	5S ribosomal RNA
trnA(UGC)*2	trnA(UGC)*2	trnA(UGC)*2	trnA(UGC)*2	tRNA-Ala
trnC(GCA)	trnC(GCA)	trnC(GCA)	trnC(GCA)	tRNA-Cys
trnD(GUC)	trnD(GUC)	trnD(GUC)	trnD(GUC)	tRNA-Asp
trnE(UUC)	trnE(UUC)	trnE(UUC)	trnE(UUC)	tRNA-Glu
trnF(GAA)	trnF(GAA)	trnF(GAA)	trnF(GAA)	tRNA-Phe
trnfM(CAU)	trnfM(CAU)	trnfM(CAU)	trnfM(CAU)	tRNA-Met
trnG(GCC)	trnG(GCC)	trnG(GCC)	trnG(GCC)	tRNA-Gly
trnG(UCC)	trnG(UCC)	trnG(UCC)	trnG(UCC)	tRNA-Gly
trnH(GUG)	trnH(GUG)	trnH(GUG)*2	trnH(GUG)*2	tRNA-His
trnI(CAU)*2	trnI(CAU)*2	trnI(CAU)*2	trnI(CAU)*2	tRNA-Ile
trnI(GAU)*2	trnI(GAU)*2	trnI(GAU)*2	trnI(GAU)*2	tRNA-Ile
trnK(UUU)	trnK(UUU)	trnK(UUU)	trnK(UUU)	tRNA-Lys
trnL(CAA)*2	trnL(CAA)*2	trnL(CAA)*2	trnL(CAA)*2	tRNA-Leu
trnL(UAA)	trnL(UAA)	trnL(UAA)	trnL(UAA)	tRNA-Leu
trnL(UAG)	trnL(UAG)	trnL(UAG)	trnL(UAG)	tRNA-Leu
trnM(CAU)	trnM(CAU)	trnM(CAU)	trnM(CAU)	tRNA-Met
trnN(GUU)*2	trnN(GUU)*2	trnN(GUU)*2	trnN(GUU)*2	tRNA-Asn
trnP(UGG)	trnP(UGG)	trnP(UGG)	trnP(UGG)	tRNA-Pro
trnQ(UUG)	trnQ(UUG)	trnQ(UUG)	trnQ(UUG)	tRNA-Gln
trnR(ACG)*2	trnR(ACG)*2	trnR(ACG)*2	trnR(ACG)*2	tRNA-Arg
trnR(UCU)	trnR(UCU)	trnR(UCU)	trnR(UCU)	tRNA-Arg
trnS(GCU)	trnS(GCU)	trnS(GCU)	trnS(GCU)	tRNA-Ser
trnS(GGA)	trnS(GGA)	trnS(GGA)	trnS(GGA)	tRNA-Ser
trnS(UGA)	trnS(UGA)	trnS(UGA)	trnS(UGA)	tRNA-Ser
trnT(GGU)	trnT(GGU)	trnT(GGU)	trnT(GGU)	tRNA-Thr
trnT(UGU)	trnT(UGU)	trnT(UGU)	trnT(UGU)	tRNA-Thr
trnV(GAC)*2	trnV(GAC)*2	trnV(GAC)*2	trnV(GAC)*2	tRNA-Val
0	0	trnV(UAC)	trnV(UAC)	tRNA-Val
trnW(CCA)	trnW(CCA)	trnW(CCA)	trnW(CCA)	tRNA-Trp

trnY(GUA)	trnY(GUA)	trnY(GUA)	trnY(GUA)	tRNA-Tyr
ycf1*2	ycf1*2	ycf1*2	ycf1*2	hypothetical protein ycf1
ycf2*2	ycf2*2	ycf2*2	ycf2*2	photosystem I assembly protein Ycf2
ycf3	ycf3	ycf3	ycf3	photosystem I assembly protein Ycf3
ycf4	ycf4	ycf4	ycf4	photosystem I assembly protein Ycf4
ycf68*2	ycf68*2	ycf68*2	ycf68*2	hypothetical protein ycf68

Note: 0 present the gene not exist in the species. *2 present two copy exist in the species.
