# Supplementary figures and images for: Comparative analysis of four Zantedeschia chloroplast genomes: expansion and contraction of the IR region, phylogenetic analyses and SSR genetic diversity assessment
Source: PeerJ. 2020 May 22;8:e9132. doi: 10.7717/peerj.9132 (PMC7247528; doi:10.7717/peerj.9132)

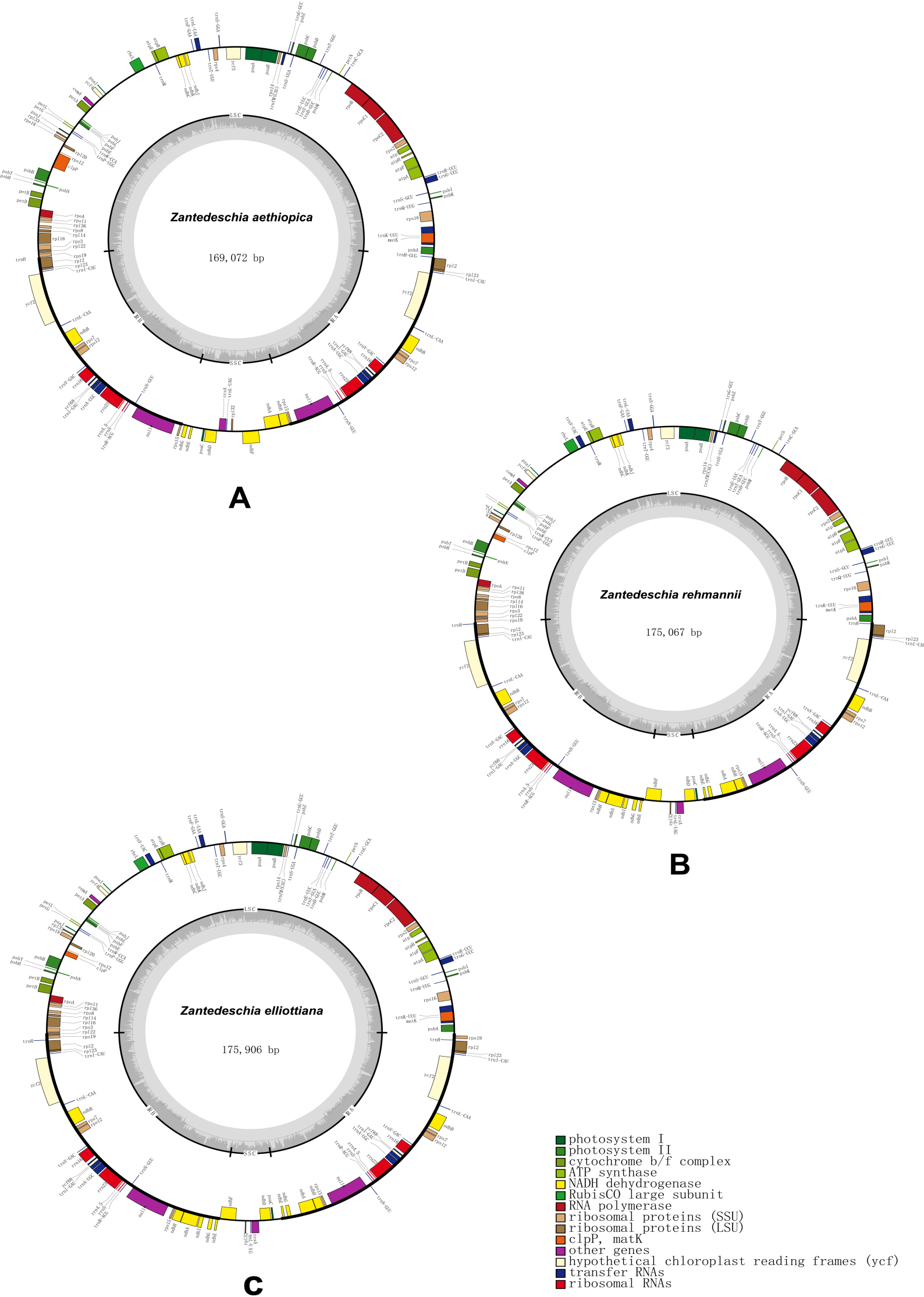

Supplement: Figure S1 [file peerj-08-9132-s004.pdf]
